# Gossip and competitive altruism support cooperation in a Public Good game

**DOI:** 10.1098/rstb.2020.0303

**Published:** 2021-11-22

**Authors:** Francesca Giardini, Daniele Vilone, Angel Sánchez, Alberto Antonioni

**Affiliations:** ^1^ Faculty of Behavioural and Social Sciences, Department of Sociology, University of Groningen, Groningen, The Netherlands; ^2^ Laboratory of Agent Based Simulation, Institute of Cognitive Science and Technology, National Research Council, Rome, Italy; ^3^ Grupo Interdisciplinar de Sistemas Complejos (GISC), Department of Mathematics, Carlos III University of Madrid, Leganés, Spain; ^4^ Instituto de Biocomputación y Física de Sistemas Complejos (BIFI), Universidad de Zaragoza, 50018 Zaragoza, Spain; ^5^ Unidad Mixta Interdisciplinar de Comportamiento y Complejidad Social (UMICCS) UC3M-UV-UZ, 28911, Leganés, Madrid, Spain; ^6^ Institute UC3M-Santander for Big Data (IBiDat), Universidad Carlos III de Madrid, 28903 Getafe, Madrid, Spain

**Keywords:** cooperation, competitive altruism, gossip, reputation, honesty

## Abstract

When there is an opportunity to gain a positive reputation, individuals are more willing to sacrifice their immediate self-interest. Partner choice creates opportunities for competitive altruism, i.e. individuals compete to be regarded as more generous and to be chosen for future partnerships. Tests of the competitive altruism hypothesis have focused so far on reputation based on direct observation, whereas the role of gossip has not been theoretically and empirically addressed. Partner choice can create an incentive to cooperate and to send truthful messages, but it can also work in the opposite direction. In order to understand the consequences of partner choice on cooperation and gossip, we designed an experimental study in which participants played a sequence of Public Goods games and gossip rounds. In our two treatments, we observed that cooperation increased when there was an opportunity to be selected, but also that cooperators sent more honest messages than defectors, and that this strategy was prevalent in the treatment in which inter-group competition was implemented. We also found evidence that participants detached themselves from the information more often when lying. Taken together, our study fills a theoretical and empirical gap by showing that partner choice increases both cooperation and honesty of gossip.

This article is part of the theme issue ‘The language of cooperation: reputation and honest signalling’.

## 1. Introduction

Reputation has been convincingly argued to be a key ingredient of cooperation in humans [1–4]. It is defined as a ‘shared evaluation that others hold about these actors with regard to one or more criteria’ [5]. Reputation allows individuals to form expectations about prospective partners’ behaviours, but it also motivates people to sacrifice their immediate self-interest in order to engage in cooperative interactions in the future. Evidence from the laboratory [2,3,6–8] and the field [9–11] shows that when indirect reciprocity is possible, knowing about someone’s reputation enables group members to preferentially interact with those who cooperate and avoid those who defect (e.g. [4,12–15]).

In a ‘market for cooperators’ [16], i.e. when partner choice is available, building a positive reputation can be seen as a long-term investment. Large groups can be seen as a market-place in which individuals would trade as buyers and sellers of cooperation in order to form the most successful coalitions [17,18]. Mutualistic theories of cooperation [19] and the competitive altruism hypothesis [20,21] pose that gaining a good reputation is essential for stabilizing cooperation, because co-operators will compete to be chosen by other co-operators, while defectors will be selected out.

Reputation-based partner choice [22,23] is based on three assumptions. First, that individuals must differ, in terms of their resources or intentions, so some of them can be better partners than others. Second, helping others can be interpreted as a signal of their quality, and last, individuals can choose with whom to interact. A growing body of experimental work shows that people are more generous when their behaviour could affect the decisions of potential partners [22], therefore supporting the competitive altruism hypothesis. A crucial tenet of the theory is that a signal of individuals’ generosity must be readily observable to others. The cooperative act is performed in front of an audience which uses this information to form a judgement about the cooperator [24]. Once this positive impression becomes shared, the actor will have a positive reputation and, because of partner choice, cooperators will strive for the best reputation by using their contributions as costly signals of cooperative intentions. However, this strategy is effective only if those signals are accurately perceived and interpreted by the receiver [25,26] otherwise the cost of producing them is not counterbalanced by any benefits. Thanks to language, humans can transcend the limits of direct observation and inform their peers about others’ behaviours.

Gossip is the cornerstone of reputation in humans, and the choice between honest and truthful information about others or lying creates the ‘gossiper’s dilemma’ [4,27]. Because language brings forward multiple opportunities for lying, gossip is usually assumed to be manipulative and unreliable, even if there is scant evidence about the actual amount of false gossip circulating in human networks. According to some scholars, gossipers would have incentives to deceive receivers in ways that benefit the gossipers themselves [28], thus derogating rivals and masking their faults. Barkow *et al*. [29] suggest that selection would have favoured our disseminating information in the interests not of objective truth but of our own success in social competition.

This view of gossip as being inherently unreliable has been recently questioned by empirical evidence indicating that the manipulative potential of gossip is less detrimental to cooperation than expected [30]. This holds true also when competition between players is introduced: even if lies become twice as frequent this has no notable effect on trust levels [31]. Similarly, modelling work [32] shows that, even in the presence of dishonest gossipers, cooperation can evolve among artificial agents playing a Public Goods game (PGG) with partner selection. The combination of privately transmitted information about other agents and partner choice drives defectors out, even in a system in which half of the population is composed of cheaters and messages are unreliable. However, previous works on competitive altruism suggest that individuals have an incentive to send dishonest signals about their own cooperative attitudes, i.e. appearing cooperative in order to later defect [21], but no testable predictions about the honesty of gossip have been developed so far.

This study contributes to the literature on cooperation and, more specifically, on the debate about the reliability of gossip by extending competitive altruism theory and suggesting that partner choice could ensure both material and informational cooperation. If cooperation is a costly signal, then reputations need to be effective in discriminating between cooperators and defectors. We hypothesize that cooperators have an incentive to be honest in order to preserve the signalling value of reputations and benefit from their previous material investment. Cooperation would go hand in hand with honest gossip because only truthful and honest information about cooperators and defectors will allow cooperators to benefit from partner choice. In a complementary manner, defectors would have an incentive to spoil the reputation system, actively contributing to its unreliability. If most of the gossip is dishonest it also becomes useless, therefore benefiting those who did not contribute because it becomes impossible to identify who the cheaters are.

In addition to lying about content, gossip can also entail a misrepresentation of the source. Previous studies on false gossip mostly focused on content manipulation, without considering how important it can be to change the source when reporting information about an absent third party. Qualifying one’s endorsement or presenting information as originating with someone else, like ‘people say’ or ‘rumour has it’, distances the speakers from the information, thereby reducing their responsibility for what is told [33,34]. Peters & Fonseca [31] show that gossip can be used as punishment, but saying that ‘someone told me’ removes that opportunity. Both the content and the source of gossip can be strategically manipulated by the gossiper [35–37], and individuals can easily lie about the content if they can attribute the information to an unknown and unverifiable source. In a cross-linguistic vignette study, Giardini *et al*. [37] show that information was more likely to be presented as indirect when it was false, thus providing evidence for preemptive action accompanying uncooperative behaviour. Source manipulation was even more frequent when there was competition between the fictional characters to access a limited resource, therefore here we expect to observe participants using an indirect source more than a direct one as a reputation management strategy, especially when lying. When intentionally misreporting someone’s behaviour, using an indirect source can be an effective strategy to avoid retaliation, because if ‘someone told you’ you cannot be held accountable for the reported information. To date, evidence about source manipulation in gossip is still scant and this study aims to provide further evidence about the antecedents and consequences of reporting information about absent third parties as first- or second-hand.

**Figure 1.**
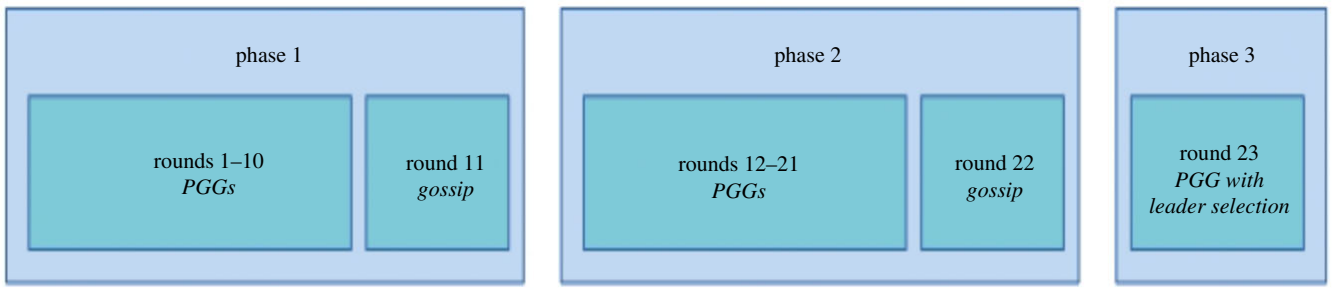
Experimental design. Phase 1 and 2 include a PGG stage of 10 rounds and a gossip stage of 5 min played in four groups of four participants each. Groups remained unchanged during each stage. Phase 3 only involves one round of a PGG with leader selection played in two groups of eight participants; this last phase is paid only to the most contributing group in the competitive treatment and to both groups in the non-competitive one. (Online version in colour.)

In order to test the relationship between competitive altruism, cooperation and the reliability of gossip we designed an experiment in which two blocks of PGG are played by groups of four participants (figure 1). Each block of 10 PGGs is followed by a gossip stage in which participants could send messages through a digital platform. For each message, they selected one or more receivers, the target and the content from a set of predefined options. We also gave participants the opportunity to choose between one of two ways of identifying the source: either ‘I know that…’ or ‘Someone told me…’. The experiment ended with a final one-shot PGG preceded by partner choice, in which two randomly selected leaders chose their group members. In the competitive treatment, only the group with the highest score in the final stage received payment for it, while players in the losing group received only the payoffs from the first two rounds. In the non-competitive treatment, both groups are rewarded. Participants were informed about every stage of the experiment, so they could decide whether to invest in reputation building. In a system in which individuals have an incentive to be chosen for future partnerships, being cooperative and being an honest gossiper can be regarded as effective strategies that, at the aggregate level, will ensure the stability of cooperation. We developed and tested three specific hypotheses about the way in which reputation-based partner choice can lead to cooperation and honest gossip (see table 1 for a summary). First, we expect to observe a higher number of high contributions in the PGG and no end-of-the-game effect, as predicted by Barclay & Willer [21], because it would work as an honest signal for future encounters. In an analogous manner, competitive altruism will motivate participants to cooperate more in phase two, where cooperation can be used as a signal of quality/intention before partner selection occurs in the last round. Second, we hypothesize that in the competitive treatment players will use gossip strategically in order to increase their chances of being selected in the last stage. This hypothesis can be further specified by contrasting the hypothesis that cooperators will preferentially use true messages in order to contribute to and maintain the reliability of gossip (H2a) with the hypothesis that they will mostly use false messages in order to ruin others’ reputations and increase their standing (H2b), as predicted by the competitive altruism theory [21]. The third and last hypothesis (H3) is about the likelihood of using the indirect source when lying, as is expected according to Giardini *et al*. [37]. Table 1 offers an overview of the hypotheses, their source and how we tested them.

**Table 1.** Overview of hypotheses.

theory-based hypotheses	source	operationalization
**H1**: Partner choice increases cooperation; **H1a**: within the same phase and **H1b**: across phases.	competitive altruism theory [20,22,38,39]	increased frequency of above-average contributions and lower amount of 0 or low contributions towards the end of the game
**H2**: Gossip is used strategically; **H2a**: cooperators will preferentially use true messages; **H2b**: cooperators will mostly use false messages.	competitive altruism theory [20]	correlation between material and informational cooperation
**H3**: Gossip is reported as coming from an indirect source more often when the content is false.	reputation management theory [5,32]	higher frequency of false messages from an indirect source

## 2. Methods

### (a) Participants in the experiment

Participants were recruited from the University of Valencia Experimental Economics Laboratory’s (LINEEX) subjects pool. One hundred and sixty students from different faculties of the University of Valencia, excluding Economics students (mean age=21.7 years; 89 females), took part in this study. They received a flat payment of 5 EUR and the opportunity to earn an additional payment ranging from 8 to 16 EUR (mean total payment=17.5 EUR). Eighty subjects, divided into five groups of 16, took part in the competitive treatment while the other 80 subjects participated in the non-competitive treatment. Laboratory experiments were conducted at LINEEX on 16 and 17 September 2015. Each experimental point was converted to real money with an exchange rate of 100 points=1 EUR. Each experimental session lasted about 45 min.

### (b) Experimental design

Each experimental session involved a group of 16 participants playing three phases. In order to ensure anonymity, participants were identified by pseudonyms, i.e. 16 moons of the solar system, that remained unchanged during the entire session. Communication between participants was not allowed and they were not informed about others’ pseudonyms. No participants were excluded from the analysis and all of the 160 registered participants completed the experiment. Two experimental treatments called Competitive (COMP) and Non-Competitive (NCOMP) were carried out. COMP and NCOMP treatments were identical except for phase 3. Participants were informed about all treatment details at the beginning of the session when reading the experimental instructions. Figure 1 shows the sequence of the three experimental phases and rounds. For the full experimental instructions, see the electronic supplementary material, section S1.

#### (i) Phase 1: Public Goods game + information exchange (gossip)

Each participant was randomly assigned to a group of four members that remained the same during the entire PGG stage. Each group completed 10 rounds of a standard PGG with a value of 1.5 as multiplication factor and a round endowment of 50 points. Participants were informed about others’ contributions and their own payoffs at the end of each round. After the PGG stage, the first gossip stage started (in the experiment we used the term ‘information exchange’ and not gossip). In this stage, participants were assigned to four gossip groups formed of four members each, selected in order to have: two members from one previous PGG group and two members from another PGG group. During this stage, participants were given the opportunity to send multiple messages to one, two, or all other members of their gossip group. Messages were displayed as a chat text through a computer interface and participants could only communicate through predefined texts. The sender, i.e. the participant who is the source of the message, can begin the message with only two predefined sentences: ‘I know that…’ or ‘Someone told me that…’, and can choose the target and the recipient of the message among the other 15 participants of the session. Then, the sender selects the content of the message between ‘cooperates’ and ‘does not cooperate’. Here follows two message examples:

Dione to Proteo: ‘I know that Temisto cooperates’.

Adrastea to Rea: ‘Someone told me that Febe does not cooperate’.

There was no limit on the number of exchanged messages but the information exchange stage was set to be 5 min long.

#### (ii) Phase 2: Public Goods game + information exchange (gossip)

Each participant was assigned to a new group of four members who remained unchanged during this PGG stage (again 10 rounds). Groups were formed in order to have combinations of two participants who had interacted before and two strangers, therefore we could control for the reliability of the content and the source. Then, another stage of information exchange played in four other different groups followed. The information exchange stage lasted 5 min, exactly like the previous one.

#### (iii) Phase 3: One-shot Public Goods game with group leaders

In the last stage, two new groups were formed through partner selection by two randomly selected group leaders. Group leaders alternatively chose seven other group members, with whom they completed a one-shot PGG with a value of 3 as multiplication factor and an endowment of 50 points. In the COMP treatment only the group contributing the most was paid for this phase, while in the NCOMP treatment both groups were rewarded according to their contributions. Group leader selection and a higher multiplication factor were designed to make partner selection salient to the participants.

## 3. Results

### (a) Gossip message classification

**Figure 2.**
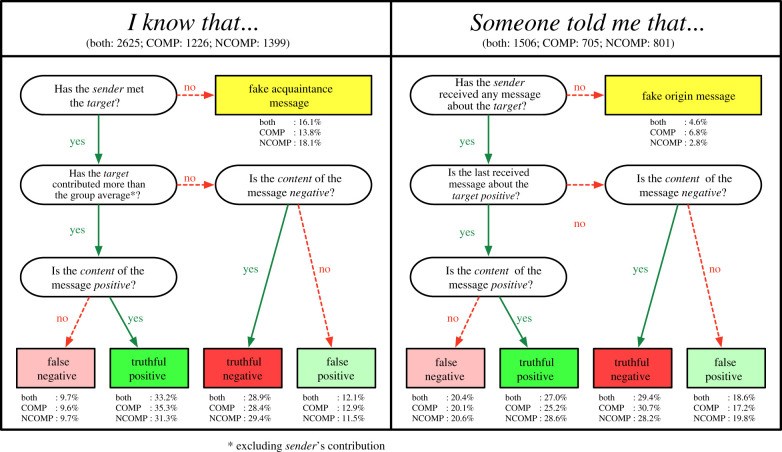
Gossip message classification. Each message is classified by its type (*I know that…*/*Someone told me that…*), content (*positive*/*negative*) and knowledge that the sender has about the target, as a fake, false or truthful message. We report the total numbers of messages for both treatments, as well as their percentages for each classification according to the message type. (Online version in colour.)

We classified each gossip message according to the scheme in figure 2. ‘I know that…’ messages are classified as fake acquaintance messages if the sender did not interact with the target before (in this case, only 6% of the time the sender also received a message about the target and we thus did not distinguish the two situations); otherwise, they are classified as false when the sender does modify the content of the message or as truthful when the sender does not. If the sender experienced that the target contributed in the PGG stage, on average, more than the group average excluding sender’s contribution, the sender should report a positive content to be considered as a truthful message, and vice versa. On the other hand, when the content of the message is inverted, we distinguish the two possible cases as false positive and false negative messages. This classification of truthful and false messages is based on sender’s group average contribution (relative classification); comparable results were obtained when considering messages to be reported as truthful positive if the target contributed on average more than half of the endowment, i.e. 25 points, in the PGG (absolute classification). We include in the electronic supplementary information, section S5, the participants’ distribution according to the relative contribution classification.

‘Someone told me that…’ messages are classified as fake origin messages if the sender did not receive any message about the target (in this case, the sender also interacted with the target during a PGG stage 26% of the time). In a similar manner, messages are then classified as false or truthful depending on whether the sender modifies the content of the last received message about the target.

We report in figure 2 the most relevant frequencies of message classification by type (‘I know that…’/‘Someone told me that…’), and treatment (for a complete overview of message frequencies, see also electronic supplementary material, section S2). We observe small, but significant, treatment effects when we compare fake acquaintance message frequencies (*χ*^2^-test: *χ* = 8.6374, d.f. = 1, **p = 0.0033) and ‘I know that…’ truthful positive message frequencies (*χ*^2^-test: *χ* = 1.9563, d.f. = 1, *p = 0.0327), having more fake acquaintance messages in the NCOMP treatment (COMP=13.8%, NCOMP=18.1%) and more ‘I know that…’ truthful positive messages in the COMP treatment (COMP=35.3%, NCOMP=31.3%). On average, participants in the COMP treatment sent more truthful positive messages as a direct source at the expenses of the fake acquaintance messages. No other remarkable differences are present when measuring treatment effects for the other kinds of single classification messages and at the group level (*χ*^2^-tests on total messages and *t*-tests on sent message frequencies by each independent group).

Furthermore, when a participant sends a ‘I know that…’ message, its content is truthful more than half of the time (both treatments, 62.1%), as well as the other type of messages ‘Someone told me that…’ (both treatments, 56.4%), with a significant difference in favour of the former type of messages (*χ*^2^-test: *χ* = 12.813, d.f. = 1, ***p = 0.0003). This result means that truthful messages are generated more often from a direct source when considering both treatments. On the other hand, this difference is more visible and statistically significant in the COMP treatment (63.7% versus 55.9%; *χ*^2^-test: *χ* = 11.154, d.f. = 1, ***p = 0.0008) than in the NCOMP treatment (60.7% versus 56.8%; *χ*^2^-test: *χ* = 3.0213, d.f. = 1, *p* = 0.0822).

Finally, to confirm our H3, ‘Someone told me that…’ messages are classified as false more frequently than direct source messages when looking at both treatments (39.0% versus 21.8%; *χ*^2^-test: *χ* = 138.51, d.f. = 1, ***p < 0.0001), as well as for the COMP treatment (37.3% versus 22.5%; *χ*^2^-test: *χ* = 47.945, d.f. = 1, ***p < 0.001) and the NCOMP treatment (40.4% versus 21.2%; *χ*^2^-test: *χ* = 91.93, d.f. = 1, ***p < 0.0001).

### (b) Individual cooperative behaviour and gossip strategy

**Figure 3.**
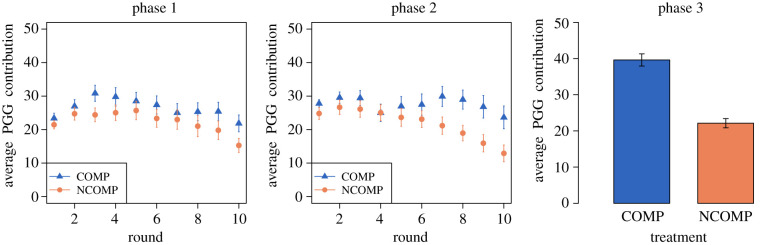
Participants’ PGG cooperative behaviour. Average group contributions in the three phases of the experiment by treatment. Error bars represent standard errors of the mean over all independent groups. (Online version in colour.)

In figure 3, we first present the results on participants’ PGG cooperative behaviour during the three phases and for the two treatments. Participants in the COMP treatment contributed more to the common pool with respect to those in the baseline NCOMP treatment. This finding is in line with previous experimental tests of the competitive altruism hypothesis [20,22,38] and with our H1a (Mann–Whitney (MW) test by individual averages: phase 1 and phase 2, *W* = 4109, **p = 0.0019; phase 3, *W* = 5235.5, ***p < 0.001; MW test by group averages: phase 3, *W* = 100, ***p < 0.001).

As predicted by the competitive altruism hypothesis, no remarkable end-of-game effect on contributions is present in the COMP treatment. In fact, participants were even more generous in the last rounds of COMP phase 2 with respect to NCOMP, when the pressure to be chosen as partners was higher and generosity could increase one’s chance of being selected during phase 3. In line with our H1b, differences on individual average contributions by phase are enhanced and statistically significant the closer participants are to the end of the experiment (MW test by individual averages: phase 1, *W* = 3834, *p = 0.0306; phase 2, *W* = 4068, **p = 0.0031).

**Figure 4.**
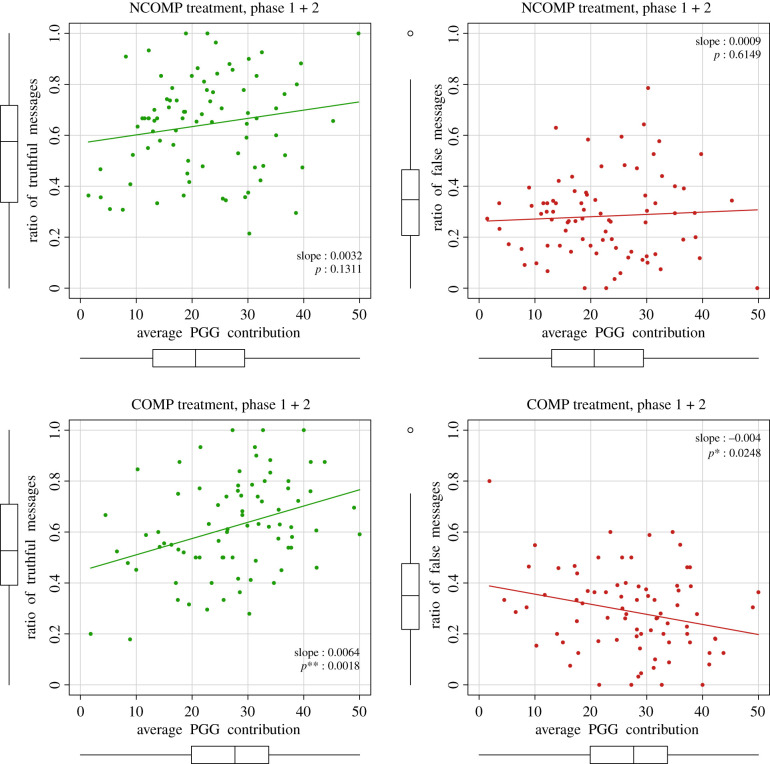
Individual cooperative behaviour and gossip strategy. Correlations between individual average PGG contribution and ratio of truthful and false messages by treatment. Each point represents a participant. Regression coefficients are reported as slopes together with their respective *p*-values using a linear regression model (OLS). (Online version in colour.)

In figure 4, we present the results at the individual level by combining participants’ contribution levels in the PGG with their gossip strategy as the ratio of truthful and false messages. Regression results can be found in table 2. Additional statistical analysis and results on participants’ behaviour can be found in the electronic supplementary material, section S3. In agreement with our H2, we find that participants use different gossip strategies depending on the treatment and phase they are in. In the NCOMP treatment, correlations between gossip and cooperation are weak, therefore there is no significant difference between gossip behaviour of highly cooperative participants and lowly cooperative ones. In the COMP treatment, instead, highly cooperative participants more frequently sent truthful messages with respect to low-cooperative ones, with a positive correlation on their contribution level. This result can be interpreted as cooperators increasing the number of truthful messages and gossip reliability to gain a better reputation and emerge from the crowd as better contributors to be chosen right before the partner selection of phase 3. For them, in accordance with our H2a, we thus found a positive effect of the competitive setting on gossip honesty. At the same time, false messages are negatively correlated to participants’ cooperation level when interacting in a competitive setting although correlation is slightly weaker.

**Table 2.** Regression results on cooperative behaviour and gossip strategy. Participants’ cooperative behaviour influence positively their ratio of truthful messages and, not significantly, their ratio of false messages. Treatment effect is not significant on the overall ratio of truthful and false messages.

	*dependent variable:*			
	ratio of truthful messages		ratio of false messages	
	(1)	(2)	(3)	(4)
average cooperation	0.004***(0.001)	0.005***(0.001)	−0.001(0.001)	−0.002(0.001)
NCOMP treatment		0.045(0.030)		−0.013(0.026)
constant	0.525***(0.038)	0.489***(0.045)	0.319***(0.033)	0.330***(0.039)
observations	159	159	159	159
*R*^2^	0.056	0.069	0.008	0.010
adjusted *R*^2^	0.050	0.057	0.002	−0.003
residual s.e.	0.187 (d.f. = 157)	0.186 (d.f. = 156)	0.161 (d.f. = 157)	0.161 (d.f. = 156)
*F*-statistic	9.288*** (d.f. = 1; 157)	5.766*** (d.f. = 2; 156)	1.318 (d.f. = 1; 157)	0.781 (d.f. = 2; 156)

*p < 0.1; **p < 0.05; ***p < 0.01.

## 4. Discussion and conclusion

If having a good reputation allows one to enter into profitable partnerships, then in a social dilemma cooperation can persist through competition with other cooperators. When direct observation of others’ deeds is not available, gossip plays a key role in discriminating between individuals who are willing and able to cooperate and those with a history of defection [4,5]. However, to date no experimental evidence about how partner choice and competitive altruism affect the reliability of gossip and its consequences on cooperation has been provided.

In an attempt to fill this gap in the literature, we examined contributions and gossip in a sequence of PGG rounds with gossip and we contrasted individuals’ behaviours in a competitive (only the most cooperative group will be rewarded) and in a non-competitive (all the groups will be rewarded) setting. Our research shows that competition to be chosen increases cooperation and, more importantly, that cooperators and defectors adopted different gossip strategies. Cooperators were more likely to report true information about other players, as opposed to defectors, and we observed a positive correlation between contributions in the game and the reliability of gossip for cooperators but not for defectors, and only in the competitive treatment.

This study contributes to the literature on reputation-based cooperation in multiple ways. First, it provides further evidence in favour of the competitive altruism hypothesis [23]. In an environment in which individuals can be rewarded for their generosity, having a positive reputation pays off and it can trigger competition to become the best possible interaction partner [21]. Like in previous studies [40,41], we also observed an escalation of prosociality due to competition for partners, with a significant increase in contributions at the end of the games and especially before partner selection took place.

Second, to the best of our knowledge, this is the first study in which specific hypotheses about gossip strategies are derived from the theory of competitive altruism and tested. If cooperation has signalling value, then investing in it is beneficial only if the gossip about cooperators is honest, and they can be reliably identified and selected for future partnerships. In a complementary way, our results show that defectors actively try to undermine the reliability of gossip by spreading false information. This difference in gossip strategies adds to the ongoing debate about the honesty of gossip [31,39,42,43] by providing supporting evidence about the fact that gossip is mostly reliable. Even when it is not, it can still support cooperation, in line with ethnographic evidence on gossip in groups and small-scale societies [44–46]. Future work is needed to investigate the boundaries of gossip reliability and the effect of contextual variables in the decision to lie and in the development of different gossip strategies. Factors like embeddedness in a network [47], or likelihood and magnitude of punishment if caught lying could be investigated.

Finally, this work contributes to the understanding of source manipulation as part of reputation management strategies. According to previous theoretical and experimental work on source manipulation [36,37], using ‘someone told me’ could be an epistemic strategic useful to lie without being punished. The way in which the source is expressed can protect the gossiper from the consequences of their misinformation, thus proving to be an important way to manage one’s reputation. In both treatments, participants used the indirect source more often when lying, a finding that indicates the relevance of linguistic features of gossip [48].

Future work on linguistic markers of gossip and on its consequences would allow the characterization of reliability not only in terms of what is told, i.e. its content, but also of the way in which the source is identified.

Theories of reputation-based partner choice that do not explain the use of gossip are incomplete because reputations emerge from people talking about others [5,49], and what is reported does not necessarily correspond to what happened. Besides demonstrating the benefit of partner choice in supporting cooperation in a Public Goods game, this study also determined that competitive altruism can explain gossip reliability and that gossip strategies are correlated with material cooperation.

## Data accessibility

The experimental data that support the findings of this study are available at: https://www.comses.net/codebases/e61c570c-dc82-4334-9f75-2d9274afb495/releases/1.0.0/.
